# Designing a Digital Toolchain for Prosthetics: a Retrospective

**DOI:** 10.33137/cpoj.v4i2.36188

**Published:** 2021-09-21

**Authors:** M. Ratto

**Affiliations:** Faculty of Information, University of Toronto, Toronto, Canada.

**Keywords:** Health Economic Framework, 3D Printing, Innovation, LMIC, Values, Prosthetics, Orthotics, Rehabilitation

## Abstract

From 2014 until 2020, I participated in the development of a novel CAD/CAM system for lower-limb prosthetic sockets for use in Lower and Middle Income Countries (LMIC) orthopaedic clinical settings. This article provides an overview of the value principles that guided that work and the ways in which we attempted to support the clinical needs of our prosthetists and others in the clinical contexts. It will highlight how the health economic framework that is key to this special issue well describes the design choices we made in order to attend to the multiple levels of concerns and stakeholders we identified as key to success.

## INTRODUCTION

From 2014 until 2020, I participated in the development of a novel CAD/CAM system for lower-limb prosthetics sockets. That project, initiated by CBM Canada, a charitable organization that supports hospitals in the developing world, and funded by corporate, non-profit, and governmental sources, was successful insofar as it developed multiple software and hardware solutions, deployed these into multiple clinical settings in different countries, and had a direct impact on the quantity and quality of prosthetic interventions in those clinics.

A non-profit organization, Nia Technologies, was founded to support this ongoing work, and has been continually funded through philanthropic donations and corporate and non-profit grants since 2015. The specifics of that work have previously been published^1-3^ and interested readers can refer to those publications for details regarding clinical outcomes, trial data, and for more information about our specific hardware and software solutions. This paper provides an overview of the work from a broader perspective, highlighting how the health economic framework, that is key to this special issue, well describes the design choices we made to attend to the multiple levels of concerns and stakeholders in the health economic framework.

## PROJECT OVERVIEW

Founded in 2015, Nia Technology is a non-profit organization that has developed and deployed a digital scanning, design and fabrication system for the production of trans-tibial prosthetic sockets and simple orthotic devices. Designed to be used in Lower and Middle Income Countries (LMIC) orthopaedic clinics, the system has been built to integrate with the current International Committee of the Red Cross (ICRC) polypropylene Prosthetic and Orthotic (P&O) system and thus concentrates only on the socket component. This system primarily makes use of a single laptop computer, modified commodity 3D scanners and printers, and custom 3D scanning and design software called NiaScan and NiaFit respectively. The resulting system, including all software and hardware costs under US$10,000, with paediatric and adult 3D printed prosthetic sockets material costs of less than $10 per unit. The system was designed to be used in LMIC contexts by orthopaedic technicians and prosthetists trained within the ICRC training programs. It has been tested and deployed in four clinics: the Comprehensive Rehabilitation Services for People with Disability in Uganda (CoRSU), the Comprehensive Community Based Rehabilitation Clinic in Tanzania (CCBRT), the Tanzania Training Centre for Orthopaedic Technologists (TATCOT) and the Cambodia School of Prosthetics and Orthotics (CSPO). For each site, Nia Technologies personnel traveled to the clinical site for one week, installing or updating equipment and carrying out three days of training with the technicians and clinicians on site. Following this, Nia Technologies provides digital support via email and teleconference. As of February 2021, the system remains in use at CoRSU, CCBRT and TATCOT.

Nia Technologies has iteratively developed a design values framework which has guided our work. Briefly, this framework is as follows:

- Design with local needs in mind as described by stakeholders in local clinical setting.
- Develop collaboratively and field-test/evaluate in target clinical settings.
- Encourage adoption and adaptation of technologies and workflow by orthopaedic technicians and clinicians in target setting.
- Manage and maintain locally (with ongoing technical support from Nia)
- Focus on benefits to local stakeholders including people with disabilities, clinicians, and hospital administrators.

Our work has been shaped by these design values some of which were first proposed based on initial research work on P&O and in consultation with experts during workshops organized early in the project^4^ and iterated as our project progressed. As this framework demonstrates, an important aspect of our work has been to emphasize local agency and ownership of the P&O process and resulting technological intervention, even when doing so increased the technological complexity of the system. For example, early on in our design process we rejected the idea of a system where the operating of the design software was outsourced to an external CAD specialist. Instead, we focused on creating design software made to emphasize and support the knowledge of the P&O professional. While we recognized the added complexity in training and in software design this decision entailed, abiding by our design principles made such a decision necessary. Our overall goal of increasing the capacities of the LMIC orthopaedic clinics therefore included not just making the process of prosthetic production more efficient, but adjacent goals that relate to the overall context of P&O work in these settings.

## HEALTH ECONOMIC FRAMING

Our values framework connects loosely to the Health Economic Framing (HEF) described in this special issue. The HEF emphasizes the need to consider the patients, prosthetist/orthotist, and payers as important stakeholders and the relevant contexts of prosthetic use, the clinical practices in which devices are made and patients engage in rehabilitation and training, and the institutions that provide the organization and funding (Table 1).

**Table 1:** Stakeholders and contexts in Health Economic Framing.

Stakeholders	Contexts
Patients	Uses of Devices
Prosthetists	Clinical and Rehabilitation practices
Payers	Health care institutions

**Figure 1:**
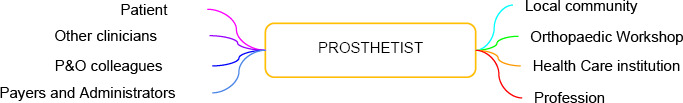
Centering of prosthetist in HEF

Our design principles - encouraged us to attend to these aspects of P&O in our design process. Creating a successful P&O intervention requires attending to the multiple relationships and needs this entails. Given that our primary users were P&O professionals working in LMIC contexts, our design principles put the orthopaedic technician and prosthetist/orthotist at the center (Figure 1).

This paper provides three specific ways our work incorporated our values framework and the key relationships highlighted above.

## EXAMPLE: RELATIONS TO LOCAL COMMUNITY CAPACITIES

The aim of Nia’s work was to speed up the process of producing and fitting a prosthetic or orthotic device within a LMIC clinical context. We designed our system to mirror and connect to the ICRC polypropylene process (first developed by the ICRC in 1979) for producing custom prosthetics and orthotics in order to make the most use of professional knowledge and skills already held by the clinicians. Our focus was on enhancing their capacity as opposed to developing a parallel process that might bypass existing professionals. Training on and access to 3D printing technology increases existing clinical capacity by providing the means for prosthetists/orthotists to make prosthetics and orthotic devices more quickly. It does not reduce or remove the need for clinicians in a clinical or rehabilitation setting.

By building on clinical skills and expertise, the technologies we developed enabled clinicians to minimise time spent on manual production and maximise time on decisions about device design, fit, and patient care. Rather than offload this work to external western partners, the 3D technology becomes another tool in the orthopaedic workshop. As an example, the team decided on using a 3D printer that is relatively easy to maintain and repair in situ, as opposed to the easiest to use printer available. This decision required the team to trust the technical capacity of the orthopaedic clinicians (as professionals already working with a range of tools and equipment), but significantly enhanced the durability of the innovation. Finally, rather than try to produce all parts of the prosthetic device, our system only replaces the custom parts; the mass-produced parts - such as connectors, pylons and feet - are cheaper if sourced through ICRC rather than 3D printed.

We applied our design values by working within the pre-existing social, professional and technical context by building on existing efforts, systems and local capacities. This stands in opposition to many other 3D printing prosthetic innovation projects that are more motivated by the application, or scaling, of a purely technological solution without the due consideration of the context that would make it viable. The former motivation offers a greater chance of implementing a long-term solution to a problem; the latter motivation can result in interventions that are not possible to sustain without the physical presence of outside short-term ‘technical experts’ (who may displace the building of more permanent capacity).

## EXAMPLE: RELATIONS TO OTHER CLINICIANS

One realization we had was a need to help our LMIC prosthetist/orthotist users represent themselves as expert to other professional groups. While highly valued for the patient results prosthetists generated in their clinical settings, ethnographic work carried out with the prosthetists/orthotists and technicians revealed a systematic lack of understanding on the expert nature of their work by others.^5^ Some of our prosthetist respondents highlighted the materiality of their work as part of the reason for this; other clinician personnel saw them mostly as ‘makers’ since they were often covered in plaster and working with the heavy vacuum pumps, grinders, and manual tools present in their workshops. The prosthetists we interviewed highlighted the ways in which the move to digital systems worked to recharacterize them as more ‘expert.’ In order to support this more fully, we encouraged the set up and design of special workstation areas, connected to but discrete from the P&O clinical setting in order to reinforce the digital aspects of their practice. Our respondents found that these settings, when witnessed by other clinical care-givers helped support the similarity of their care-giving practice to other experts in the hospital.

Here, our design work again extended beyond the specific digital scanning, design, and printing tasks to incorporate the prosthetists‘/orthotists’ relations to others in the clinical setting. Equally, our design work was not just about the software and hardware that we produced, but also included the settings in which it operated. Again, successful adoption of our system was supported by our understanding the full work flow and the complex health frameworks in which this P&O work was situated.

## EXAMPLE: RELATIONS TO FUNDERS AND PAYERS

Finally, a key aspect of the development work was in generating an innovation which helped support the overall work of the clinic and the funders who provided the resources necessary for its operation. In the LMIC contexts in which we deployed our solution, this is often non-profit and charitable organizations. For all new innovations, it is important to build up an evidence base to ‘prove’ the efficacy of the innovation, especially since it integrated technology which is new and relatively unfamiliar to the sector. Therefore, a key aspect of our work has been continual clinical evaluation of our solutions, ultimately resulting in testing over 200 patients at four clinical sites in three different countries. The goal of these trials was to be able to make statistically significant claims about the 3D printed device with regards to durability, fit, accuracy and overall use when directly compared to traditional devices. Nia set this goal for multiple reasons including both functional and symbolic needs.

Functionally, we needed to more fully evaluate and improve our processes in order to validate the (at the time) novel use of 3D printing in lower-limb prosthetics. Symbolically, our continued investment and engagement in collaborative trials with the hospitals demonstrated Nia’s commitment to improving the clinical process to the clinicians and other stakeholders, thereby strengthening key relationships. In particular, funders of clinical P&O work required clinical evidence in order to continue to support this work. We also found that the trials served to draw new funding into the orthopaedic clinic, supporting attention and interest in an area of work that required additional investment.

As in the above cases, we took the need to link P&O work to funders and payers into consideration as we developed and deployed our software and hardware interventions. In particular, we worked directly with the LMIC hospitals to develop forms of survey and reporting which served to provide clinical oversight and evaluation of the capacities and problems with our solutions, but also resulted in two additional forms of evidence. First, and most importantly, we generated economic evidence that used very situated material, labour, and support costs to define the potential economic benefits of our system. These typically required deep dives into the specifics of the LMIC clinics, including working with the clinical staff to access actual numbers associated with orthopaedic clinical work. This often required putting relatively complex economic stories together, where the economics of the clinic including device production, rehabilitation, and education costs were connected to more general expenses associated with housing and transportation of prosthetic patients and their families during treatment. These stories assisted the hospitals in funding the use of our systems, but also often resulted in new forms of funding for the orthopaedic clinic and in some cases the hospital itself.

## DISCUSSION

The above story and examples may make it seem that the processes through which Nia Technologies developed and deployed a new 3D scanning, design, and printing solution were straight-forward and clear, and the results guaranteed. Nothing could be further from the truth. The work did not follow a straight-forward path, nor has acceptance of our solutions been complete. While our solution does continue to be utilized in some of the clinical settings I mentioned, in at least one of them, it was not adopted despite concerted efforts. However, the lack of adoption of Nia’s solutions and, of 3D printing in P&O more generally, should not be understood as simply an issue in the capacity of 3D printing and digital systems to meet the needs of P&O. Nor should this slowness be understood as the result of recalcitrant P&O professionals unwilling to adopt novel tools. While it is true that convincing orthopaedic clinicians of the merits and potential of 3D technologies has required a longer evidentiary process than we initially assumed, adoption or its lack is better understood as ‘lack of fit’ into the full framework of P&O work A key aspect that is often undervalued in contexts of emerging technologies like 3D printing and CAD is the provision of training and support that must attend actual implementation. We found that both in-person and distributed forms of support were key to successful adoption, including periodic connections to other institutional actors outside the direct orthopaedic context, such as hospital administrators. Developing innovations in this space requires attending to the full socio-technical context of P&O work, including the various relationships well described in the Health Economic Framework which is the focus of this special issue.

Key to overcoming this challenge was helping the profession understand that 3D printing technologies would not replace clinicians with CAD designers in Canada (or elsewhere), nor would their craft and expertise be transformed into manual labour with the remainder being done automatically by a computer or by a different set of professional CAD designers. Retaining the clinician at the centre of the design and production process by integrating 3D technologies into the profession remains a major aspect of Nia’s work. In doing so, our goal is to strengthen the profession and assist P&O professionals in better communicating the complexity of their care-giving work to others within their clinical contexts. To be clear, the need and clinical importance of strengthening and clarifying the role of the prosthetist was not initially apparent to us. Instead, the surfacing of these aspects occurred through our sustained connection to clinical contexts and the qualitative data-gathering and analysis we carried out as part of an ethnographic process. Finally, investment in professional development, quality and patient care takes time and significant resources. Technology funders in particular have grown accustomed to a specific notion of scale, i.e. that technology innovations can be scaled quickly, leading to quick and large scale results, providing opportunities for funders that require a relatively short-term commitment. Innovation within P&O as in any health care context requires more than just material and technical development, it also requires analysis of the deep web of relations through which the profession provides its care.

## CONCLUSION

Media coverage of prosthetics and 3D printing has often focused on the impacts and value to the patient, while ignoring the prosthetic profession and clinical contexts of this work. Personally, I continue to receive requests for ‘legs’ from people all over the world, including many requests from prosthetic wearers here in Canada who, typically for economic reasons, have difficulty securing devices for their use. Such requests, although well-meaning, misconstrue the role of new technical innovations like 3D printing in the established field of prosthetics and the socio-technical contexts that guarantee clinically valid results for prosthetic users. Too often, engineers and innovators from outside the field of P&O help reinforce this mistake, building systems intended to ‘transform the field’ but which typically fall flat. This paper describes an alternative innovation approach, that the team that participated in the Nia technologies project ‘backed into’ as we became more knowledgeable about P&O. The values principles we developed and the HEF relations to which we attended account for our relative successes; our inability in some cases to fully support the complexities of local relations equally accounts for a lack of adoption. The goal of this brief article has been to anecdotally describe the ways in which we attempted to incorporate a more complex understanding of P&O contexts as part of our project in the hopes that these experiences will provide support for others seeking to innovate.

## CALL TO ACTION

Many technically sophisticated tools exist that can benefit the P&O community. When adoption is slow, prosthetists and clinics are often blamed, with innovators claiming that the issue is primarily the conservative nature of the P&O discipline. Our experiences developing Nia Technologies’ digital toolchain for lower-limb prosthetic sockets highlights the ability of P&O clinics and clinicians to be early adopters of new technologies if and when the solutions being developed address the many needs and stakeholders that exist in the field. I encourage all innovators in P&O to first, incorporate a deep dive into specific P&O contexts (public health, private providers, LMIC clinics, developed world contexts, etc.) prior to developing their initial solution requirements, and second, to plan to work directly and iteratively with clinicians in their planned context of deployment. In doing so, P&O innovators will be able to better address the complexity of the P&O context, including the stakeholders identified within the Health Economic Framework.

## DECLARATION OF CONFLICTING INTERESTS

During the time that my research on prosthetics and digital systems was being carried out, I was paid a consultancy fee by Nia. At the time of writing this paper, I was no longer receive any financial fees or incentives from Nia.

## SOURCES OF SUPPORT

I have been Chief Science Officer of Nia Technologies, the non-profit organization described in this article since 2015. This role has involved consultancy payment. The writing of work was not directly supported by Nia, nor any of the grants Nia uses for current or past support.

## AUTHOR SCIENTIFIC BIOGRAPHY

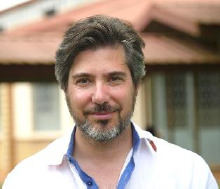



**Matt Ratto** is an Associate Professor in the Faculty of Information at the University of Toronto and the Bell University Labs Chair in Human-Computer Interaction. His current work explores the co-design and adoption of new digital technologies in health care. He coined the term “critical making” in 2007 to describe work that combines humanities insights and engineering practices and has published extensively on this concept. A current project involves the development of a cost-effective software and hardware toolchain for the scanning, design, and 3D printing of lower-limb prostheses for use in the developing world. This work is being carried out in partnership with Hope and Healing International, and rehabilitation hospitals in Canada, Uganda, and Tanzania.

## References

[1] Ratto M, Qua Hiansen J, Kaweesa M, Taremwa J, Heang T, Kheng S, et al. An international multi-center clinical study: gauging patient experience with digitally designing, 3D trans-tibial prosthetic devices compared to manually produced prosthetic devices. International Society for Prosthetics and Orthotics World Congress (ISPO), 2017, Capetown, SA, May 8-11, 2017.

[2] Schmidt R, Coons G, Chen V, Gmeiner T, Ratto M. 3D-printed prosthetics for the developing world. InSIGGRAPH 2015: Studio 2015. DOI: 10.1145/2785585.2792535

[3] Ratto M, Qua Hiansen J, Marshall J, Kaweesa M, Taremwa J, Heang T, et al. An International, Multicenter Field Trial Comparison Between 3D-Printed and ICRC-Manufactured Transtibial Prosthetic Devices in Low-Income Countries. J Prosthet Orthot. 2021;33(1): 54-69. DOI: 10.1097/JPO.0000000000000349

[4] Record, M. Ratto, A. Ratelle, A. Ieraci and N. Czegledy, “DIY Prosthetics Workshops: ‘critical making’ for public understanding of human augmentation,” 2013 IEEE International Symposium on Technology and Society (ISTAS): Social Implications of Wearable Computing and Augmediated Reality in Everyday Life, 2013, 117-125, DOI: 10.1109/ISTAS.2013.6613110

[5] Southwick D. Expertise in the Age of Digital Fabrication [Doctoral Thesis]. University of Toronto. 2019; Available from: https://tspace.library.utoronto.ca/handle/1807/95985

